# Impact of recanalization of chronic total occlusion on left ventricular electrical remodeling

**DOI:** 10.1111/pace.13691

**Published:** 2019-04-25

**Authors:** Kennosuke Yamashita, Wataru Igawa, Morio Ono, Takehiko Kido, Toshitaka Okabe, Naoei Isomura, Hiroshi Araki, Masahiko Ochiai

**Affiliations:** ^1^ Division of Cardiology and Cardiac Catheterization Laboratories Showa University Northern Yokohama Hospital Kanagawa Japan

**Keywords:** 3D electroanatomical mapping, chronic total coronary artery occlusion, electrical remodeling, left ventricle, percutaneous coronary intervention

## Abstract

**Background:**

Successful percutaneous coronary intervention (PCI) for chronic total occlusion (CTO) is associated with reduction of cardiac mortality, as well as reducing fatal ventricular arrhythmias. The aim of this study was to evaluate the effect of recanalization of CTO on endocardial left ventricular voltages by paired electrophysiological studies.

**Methods:**

Sixteen consecutive patients who underwent PCI for *de novo* CTO lesions were included. High‐density mapping was performed during sinus rhythm before and 8 months after PCI. According to the amplitude of bipolar electrograms, the left ventricular endocardium was classified into a preserved normal voltage (>1.5 mV), border zone (0.5–1.5 mV), and dense scar areas (<0.5 mV).

**Results:**

The border zone area had a significant positive correlation with CTO length, as well as a significant negative correlation observed in the preserved voltage region. In the successful PCI patient, the median dense scar area did not change significantly (reported as [median difference: 95% confidence interval]) between baseline and after PCI (0.1 cm^2^: –2.8 to 2.9). However, the area of the border zone decreased (–10.5 cm^2^: –16.8 to –4.1) and the preserved voltage area increased significantly (19.2 cm^2^: 7.7–30.6). In addition, successful PCI was related to slight, but significant, increase in the amplitude of unipolar and bipolar voltage (1.55 mV: 0.88–3.33, 0.23 mV: 0.08–0.36).

**Conclusions:**

Recanalization of CTO may promote reverse electrical remodeling in the border zone of the left ventricle, without affecting the dense scar tissue.

## 1 INTRODUCTION

Chronic total occlusion (CTO) is very common in patients with coronary artery disease and has a reported prevalence of 20–50% among patients referred to the catheterization laboratory with ischemic symptoms.^1^, ^2^ CTO is associated with a decrease in the left ventricular ejection fraction and is an independent predictor of the occurrence of ventricular tachycardia (VT) during follow‐up with an adverse impact on long‐term mortality.^3^, ^4^, ^5^ Several studies have shown that successful recanalization of CTO may significantly reduce angina symptoms, as well as improve the mortality rate and risk of major adverse cardiac events.^6^, ^7^, ^8^, ^9^ It has also been reported that successful percutaneous coronary intervention (PCI) for myocardial infarction improves cardiac electric stability but no data were previously published about a potential effect by CTO revascularization on electrical stability.^10^, ^11^ In contrast to nonischemic cardiomyopathy, arrhythmias in ischemic cardiomyopathy are frequently related to the endocardial substrate, with fewer patients needing epicardial access to treat VT.^12^, ^13^ However, no electrophysiological data have been published regarding the influence of recanalization of CTO on left ventricular electrical remodeling. Therefore, this study was performed to assess the impact of successful PCI for CTO on left ventricular electrical remodeling in patients undergoing paired three‐dimensional electroanatomical mapping studies.

## 2 METHODS

### 2.1 Study population

**Figure 1:**
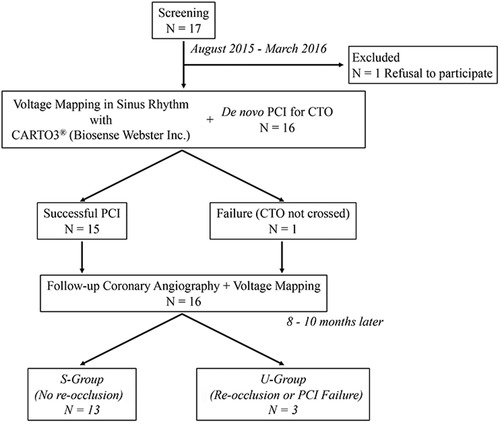
Disposition of the patients. The 16 patients were divided into S‐group (n = 13) and U‐group (n = 3). CTO = chronic total occlusion; PCI = percutaneous coronary intervention; S‐group = patients with successful PCI and no restenosis; U‐group = patients with unsuccessful PCI or with reocclusion during the follow‐up period

This prospective cohort study was conducted at the Showa University Northern Yokohama Hospital. We prospectively identified 17 consecutive patients undergoing a PCI for *de novo* native coronary artery CTO at our institution during the period from August 2015 to March 2016. The eligibility criteria are displayed in Figure 1. CTO was defined as a lesion causing complete interruption of blood flow (Thrombolysis In Myocardial Infarction flow grade 0) for an estimated duration of at least 3 months.^14^ One patient refused to participate in this study, while the other 16 gave written informed consent and were enrolled. All participants underwent segmental endocardial electroanatomical mapping of the left ventricle just before PCI and 8–10 months after intervention. Follow‐up angiography and electroanatomical mapping were performed at the same timing regardless of the outcome of PCI.

### 2.2 Computed tomography (CT) protocol

All patients underwent coronary CT within 3 months before PCI. A dual‐source CT system (Somatom Definition; Siemens Medical Solutions, Forchheim, Germany) was used with the following settings as described previously: detector collimation 64 × 0.625 mm, table feed 19.7 mm/s, 0.17 helical pitch (beam pitch), rotation time 280 ms, tube current 370 mAs, and voltage 120 kVp. The scanning time ranged from 6 to 8 seconds. Raw scan data were reconstructed using 75% of the RR interval or the applicable optimal phase. A bolus dose of contrast medium (iohexol; Omnipaque, Daiichi‐Sankyo Pharmaceutical, Tokyo, Japan) containing 350 mg iodine/mL was injected at a volume of 0.6 mL/kg within 9 seconds. In all patients, a β‐blocker (bisoprolol fumarate: 2.5 mg) was administrated orally 1 hour before CT scanning and nitroglycerin (0.3 mg) was given just before scanning. Reconstructed CT scans were transferred to a workstation for postprocessing (Ziostation; Amin, Tokyo, Japan). The CTO segment was identified visually in long‐axis and short‐axis views by using curved multiplanar reformation (cMPR) and comparison with proximal reference segments. The total length of occlusion was measured on cMPR images from the proximal to distal margins of the occluded segment, which was identified by loss of luminal continuity.

### 2.3 Angiography

Quantitative assessment was performed with an automated edge detection system (CASSII; PieMedical, Maastricht, The Netherlands). Images were analyzed by an independent observer who was not involved in the study to avoid bias. The length of each occlusion was measured from the proximal site of obstruction to the distal site of retrograde filling from contralateral collaterals by using a simultaneous bilateral injection technique, from the site where filling of bridging collaterals commenced to that where the distal vessel was clearly visualized, or from the length of the lesion visible after guidewire crossing. Collateral flow was graded according to Rentrop's classification.^15^ Other variables such as calcification, tortuosity, bridging collaterals, and stump morphology were assessed according to standard definitions.^16^

### 2.4 PCI

The indications for recanalization of CTO lesions were based on current guidelines for myocardial revascularization and stable coronary artery disease.^17^, ^18^ Baseline symptoms were assessed according to the Canadian Cardiovascular Society and New York Heart Association classifications. Recanalization of CTO was performed via the antegrade and/or retrograde approach using contemporary techniques, including double injection, dedicated wires, and microcatheters. PCI was defined as successful when complete restoration of antegrade blood flow was achieved (Thrombolysis In Myocardial Infarction flow grade 3) with <30% residual diameter stenosis. All procedures were done by a single experienced operator (M.O.). Oral administration of aspirin was started prior to the procedure. Following sheath insertion, bolus doses of unfractionated heparin (150 units/kg) were administered during the procedure to maintain an activated clotting time (ACT) of 250–300 seconds, with the ACT being measured both before and during PCI. Contraindications to PCI included intolerance of aspirin or ticlopidine and scheduled noncardiac surgery. After PCI, clopidogrel (75 mg/day after a loading dose of 300 mg) was added to aspirin and dual antiplatelet therapy was continued until follow‐up coronary angiography.

### 2.5 Electroanatomical mapping

All procedures were done under conscious sedation and performed by a single operator (K.Y.). After a 7‐or 8‐Fr‐long sheath (45 cm) was placed in the femoral artery, the left ventricle was accessed via a retrograde aortic approach. The CARTO default filter settings were used for recording bipolar signals, including a 30‐Hz high‐pass filter, a 500‐Hz low‐pass filter, and display at 200 mm/s. Unipolar signals were also recorded between the each electrode of a mapping catheter or the distal tip of the ablation catheter and the Wilson central terminal, including a filter at 1–240 Hz, and displayed at 200 mm/s. Prior to PCI, detailed CARTO electroanatomical mapping of the left ventricle was performed during sinus rhythm in all patients. Electroanatomical mapping was done with a multipolar mapping catheter (PentaRay^®^, Biosense Webster, Diamond Bar, CA, USA), which was selected for this study because it has 1‐mm electrodes with a short interelectrode distance (2 mm) when used in bipolar mode. Adequate catheter contact with the ventricular wall was confirmed by fluoroscopic guidance. An electrode catheter with a 4‐mm tip (NAVISTAR^®^, Biosense Webster) was only used for mapping when the PentaRay^®^ catheter could not do mapping during sinus rhythm without premature ventricular beats. All electrograms were obtained in sinus rhythm and were manually reviewed to exclude noise, artifacts, or premature ventricular contractions.

**Figure 2:**
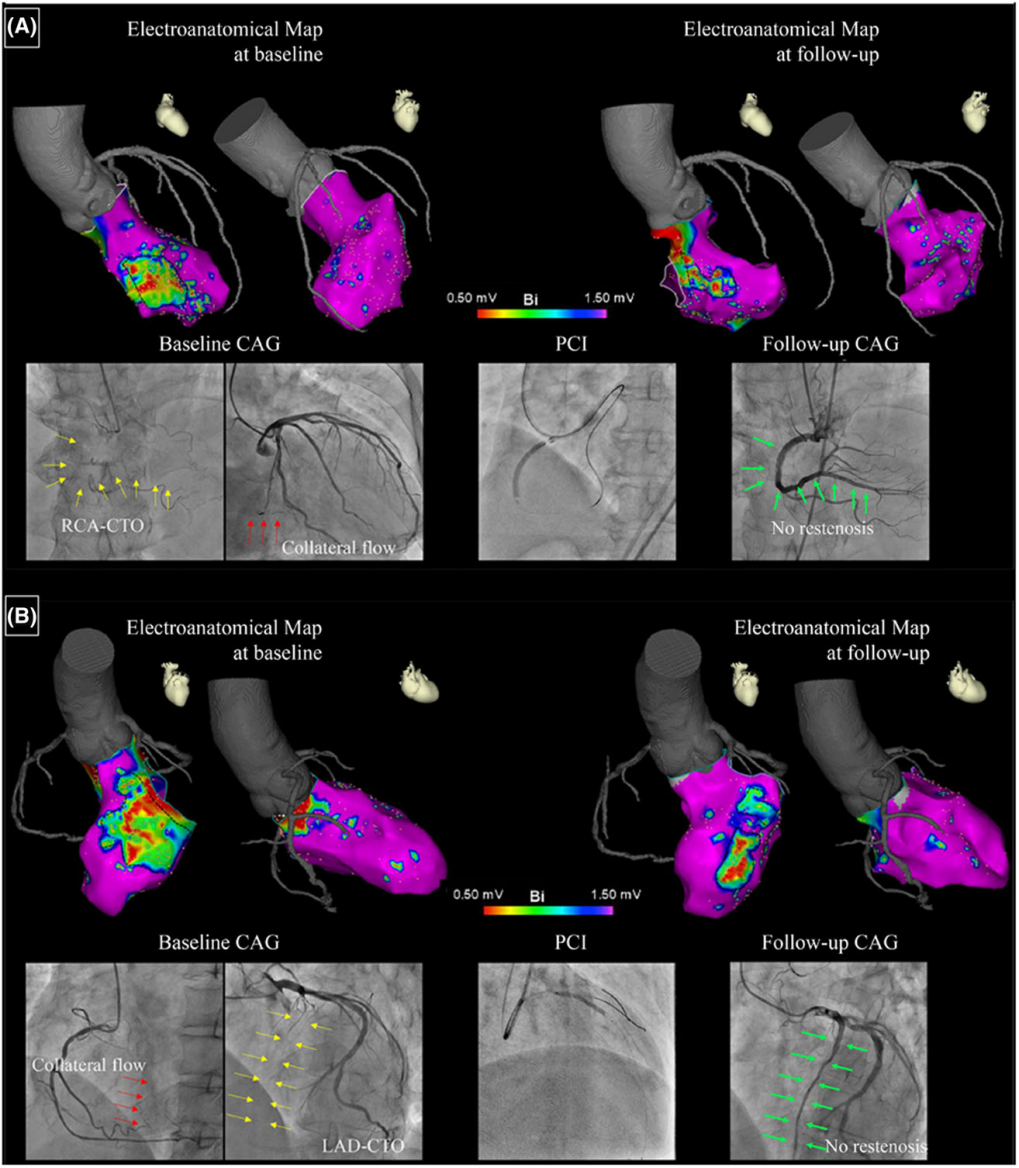
Panels A and B show examples of voltage maps and coronary angiograms at baseline and follow‐up in two patients. In the voltage maps, the preserved voltage region (>1.5 mV) is purple and the dense scar region (<0.5 mV) is red. The border zone (0.5–1.5 mV) is primarily shown as blue to orange. On baseline angiograms, the culprit CTO is indicated by a yellow arrow and the collateral is shown by a red arrow. On follow‐up angiograms, the green arrow indicates successful recanalization of the CTO. CAG = coronary angiography; CTO = chronic total occlusion; LAD = left anterior descending artery; PCI = percutaneous coronary intervention; RCA = right coronary artery [Color figure can be viewed at http://wileyonlinelibrary.com]

After endocardial mapping, registration of CT data was performed using the CARTO‐Merge software. Using the peak‐to‐peak voltage amplitude on the bipolar electrograms, left ventricular regions were defined as having a preserved voltage (>1.5 mV), a border zone voltage (0.5–1.5 mV), or a dense scar voltage (<0.5 mV).^19^, ^20^ Late potentials (LPs) were also defined as low‐voltage electrograms (<1.5 mV) showing a single or multiple continuous delayed electrical components, separated from the local ventricular electrograms by at least 20 ms and recorded after the surface QRS end.^21^ In addition, based on the threshold values of unipolar voltage amplitude,^22^ left ventricular lesions were also divided into low UNI area (≤8.27 mV) and normal UNI area (> 8.27 mV). Mapping was performed by mainly targeting the low‐voltage regions (<1.5 mV), while sufficient sampling was done elsewhere to obtain a fill threshold of 15 mm. All mapping points that were determined to be located >5 mm from the LV endocardial geometry were considered to show poor contact and were excluded from analysis. Areas within 10 mm of the aortic valve and mitral valve were also excluded from assessment. The areas of the three regions defined according to bipolar amplitude were measured by using the standard surface area measurement tool of the CARTO system and the total area was calculated automatically (Figure 2).

### 2.6 Statistical methods

Results were assessed with the JMP^®^ software (SAS Institute, Cary, NC, USA). Categorical data were expressed as frequencies and were compared with Pearson's χ^2^ test and Fisher's exact test. Continuous data were presented as median (first quartiles, third quartiles). Shapiro‐Wilk test was used to assess normality. Comparison of normally distributed variables between groups was performed by an independent‐sample or paired *t*‐test, as appropriate. Nonnormally distributed variables were compared by using the Wilcoxon matched‐pairs signed‐rank test for paired replicates. The difference was reported as median difference (95% confidence interval). The association between the surface area of each voltage region on endocardial mapping and the length of the CTO measured by CT or coronary angiography was assessed by univariate linear regression analysis. In all analyses, a probability (P) value of <0.05 was considered to indicate statistical significance.

### 2.7 Ethical considerations

This study was carried out according to the principals of the Declaration of Helsinki and the protocol was approved by the SHOWA University Clinical Research Review Board. All experiments were performed in accordance with relevant guidelines and regulations and patients’ records and information were anonymized and deidentified before analysis. Written informed consent to participation was obtained previously from all the included patients. The trial was registered at http://www.umin.ac.jp/ctr/index.htm (trial identifier: UMIN000033618).

## 3 RESULTS

Of the 16 patients who consented to participate in this study, the guidewire failed to cross the CTO in one patient. In two of the remaining 15 patients, in‐stent occlusion occurred during the follow‐up period. Accordingly, we divided the subjects into two groups: (1) 13 patients with successful PCI and no restenosis (S‐group) and (2) three patients with unsuccessful PCI or with re‐occlusion during the follow‐up period (U‐group) (Figure 1).

Assessment of baseline clinical characteristics revealed no significant differences between the two groups in terms of age, gender, body mass index, and medical history (Table 1). Coronary angiography and CT findings are listed in Table 2. There were no significant differences in the CTO characteristics, length, and procedure. While the number of stents used was higher in the successful group, there was one primary PCI failure in the U‐group.

**Table 1:** Baseline clinical characteristics

	Total N = 16	U‐group N = 3	S‐group N = 13	P‐value
Age, years	66.0 (59.8, 69.5)	65.0 (55.0, 67.0)	67.0 (60.5, 72.0)	0.41
Male gender	15 (93.8)	3 (100.0)	12 (92.3)	1.0
Height, cm	166.5 (163.1, 171.0)	169.0 (166.6, 171.6)	164.5 (160.8, 170.0)	0.32
Body weight, kg	66.6 (62.9, 74.5)	70.5 (67.4, 78.9)	65.5 (62.5, 72.6)	0.29
Body mass index, kg/m^2^	24.6 (23.3, 26.0)	24.7 (24.3, 26.8)	24.5 (23.1, 25.9)	0.46
Ejection fraction, %	38.3 (32.5, 46.3)	37.8 (30.6, 46.8)	38.7 (32.9, 46.1)	0.86
Hypertension	14 (87.5)	3 (100.0)	11 (84.6)	1.0
Dyslipidemia	14 (87.5)	3 (100.0)	11 (84.6)	1.0
Current smoker	6 (37.5)	2 (66.7)	4 (30.8)	0.50
Diabetes mellitus	6 (37.5)	1 (33.3)	5 (38.5)	1.0
Chronic kidney disease	4 (25.0)	1 (33.3)	3 (23.1)	1.0
Prior myocardial infarction	3 (18.8)	1 (33.3)	2 (15.4)	0.49
Multivessel disease	4 (25.0)	1 (33.3)	3 (23.1)	1.0
No. of diseased vessels	1 (1, 2)	1 (1, 2)	1 (1, 2)	0.73
Previous ICD	2 (12.5)	1 (33.3)	1 (7.7)	0.35

*Note*: Numbers show the median (first quartiles, third quartiles) or number of patients (%). ICD = implanted cardioverter defibrillator; PCI = percutaneous coronary intervention; S‐group = patients with successful PCI and no restenosis; U‐group = patients with unsuccessful PCI or with reocclusion during the follow‐up period.

**Table 2:** Angiographic and CT findings

	Total	U‐group	S‐group	
	n = 16	n = 3	n = 13	P‐value
CTO length (Angio), mm	29.6 (20.0, 37.6)	28.8 (23.6, 52.6)	30.4 (18.7, 37.2)	0.50
CTO length (CT), mm	30.7 (22.1, 38.9)	29.9 (26.4, 56.8)	31.4 (20.6, 38.1)	0.47
Calcification	6 (37.5)	1 (33.3)	5 (38.5)	1.0
Tortuosity	3 (18.8)	2 (66.7)	1 (7.7)	0.10
Rentrop > 2	14 (87.5)	2 (66.7)	12 (92.3)	0.40
Bridging collateral	3 (18.8)	1 (33.3)	2 (15.4)	0.50
Abrupt stump	4 (25.0)	1 (33.3)	3 (23.1)	1.0
No. of stents	3 (2, 3)	1 (0, 3)	3 (2, 3)	0.05
Stent diameter, mm	3.0 (2.5, 3.0)	2.4 (2.3, 2.5)	3.0 (2.5, 3.0)	0.15
Total stent length, mm	89.5 (61.8, 106.5)	32.0 (0, 109.0)	91.0 (65.0, 104.0)	0.11
Retrograde approach	8 (50.0)	1 (33.3)	7 (53.8)	1.0
Sheath size 7Fr/8Fr	9 (56.3)/7 (43.8)	2 (66.7)/1 (33.3)	7 (53.9)/6 (46.2)	1.0
IVUS used	16 (100.0)	3 (100.0)	13 (100.0)	1.0
Follow‐up period, days	239 (208, 260)	262 (259, 367)	231 (203, 246)	0.24

*Note*: Numbers show the median (first quartiles, third quartiles) or number of patients (%). Angio = coronary angiography; CT = computed tomography; CTO = chronic total occlusion; IVUS = intravascular ultrasound; PCI = percutaneous coronary intervention; S‐group = patients with successful PCI and no restenosis; U‐group = patients with unsuccessful PCI or with reocclusion during the follow‐up period.

Detailed electroanatomical findings of each subject with the difference of the variables before and after PCI is shown in Table 3. The median number of electroanatomical mapping points in all subjects was 1,497 (1,423, 1,552) for the baseline study and 1,425 (1,325, 1,486) for the follow‐up study. Similarly, no significant difference of mapping points before and after PCI was found in the S‐group. At baseline and follow‐up, there was no significant difference of the total left ventricular surface area in each group. When we assessed the changes in each electrical region, the preserved voltage area increased significantly (14.0 cm^2^ [2.7–25.2]) in all subjects while no statistically significant changes were observed about dense scar and border zone areas (Table 4). In the U‐group, there were no significant changes at each region after PCI. On the other hand, the area of the border zone showed a significant decrease from baseline in the S‐group. Corresponding to this reduction of the border zone region, the area of the preserved voltage region increased after PCI in the S‐group. Similarly, while the low UNI area did not change significantly in all patients, the S‐group showed the significant reduction of low UNI area. Moreover, we investigated the LPs area but the size of the LP areas did not change significantly regardless of PCI result.

**Table 3:** Electrophysiological data of each subject

		Area before PCI, cm^2^ (%)				Area after PCI, cm^2^ (%)				Difference (after – before PCI), cm^2^		
Subject	PCI success	Dense scar	Border zone	Preserved voltage	Total	Dense scar	Border zone	Preserved voltage	Total	Dense scar	Border zone	Preserved voltage
1	Yes	14.5 (5.4)	62.7 (23.4)	190.8 (71.2)	268.0	12.1 (5.0)	46.8 (19.4)	182.3 (75.6)	241.2	−2.4	−15.9	−8.5
2	Yes	9.0 (3.6)	48.6 (19.5)	191.7 (76.9)	249.3	10.8 (4.8)	38.4 (17.1)	175.2 (78.1)	224.4	1.8	−10.2	−16.5
3	Yes	11.4 (3.8)	52.6 (17.5)	236.3 (78.7)	300.3	11.4 (4.2)	34.1 (12.6)	224.9 (83.2)	270.4	−0.1	−18.5	−11.5
4	Yes	47.6 (16.8)	78.3 (27.6)	157.7 (55.6)	283.6	34.2 (13.4)	39.8 (15.6)	181.2 (71.0)	255.2	−13.4	−38.5	23.5
5	No	27.5 (11.2)	58.5 (23.8)	159.7 (65.0)	245.7	30.5 (13.8)	56.4 (25.5)	134.2 (60.7)	221.1	3.0	−2.1	−25.5
6	No	53.3 (23.4)	90.0 (39.5)	84.6 (37.1)	227.9	63.2 (25.2)	109.3 (43.6)	78.2 (31.2)	250.7	9.8	19.3	−6.3
7	Yes	31.8 (12.4)	50.8 (19.8)	173.8 (67.8)	256.4	33.6 (11.9)	33.3 (11.8)	215.2 (76.3)	282.1	1.8	−17.5	41.4
8	Yes	26.8 (9.6)	49.1 (17.6)	203.1 (72.8)	279.0	28.2 (9.2)	46.6 (15.2)	232.0 (75.6)	306.8	1.5	−2.5	28.9
9	No	13.6 (6.4)	28.1 (13.2)	170.9 (80.4)	212.6	16.6 (7.1)	40.7 (17.4)	176.6 (75.5)	233.9	3.0	12.6	5.6
10	Yes	15.6 (5.9)	35.4 (13.4)	213.5 (80.7)	264.5	11.9 (4.1)	33.2 (11.4)	245.9 (84.5)	291.0	−3.7	−2.3	32.4
11	Yes	34.2 (15.3)	50.1 (22.4)	139.4 (62.3)	223.7	35.4 (14.4)	43.8 (17.8)	166.8 (67.8)	246.0	1.2	−6.3	27.5
12	Yes	23.3 (8.5)	41.7 (15.2)	209.2 (76.3)	274.2	27.7 (9.2)	38.3 (12.7)	235.6 (78.1)	301.6	4.4	−3.4	26.4
13	Yes	30.3 (11.6)	58.9 (22.6)	171.6 (65.8)	260.8	30.1 (10.5)	53.4 (18.6)	203.4 (70.9)	286.9	−0.1	−5.6	31.8
14	Yes	33.3 (14.4)	44.9 (19.4)	153.1 (66.2)	231.3	38.7 (15.2)	45.3 (17.8)	170.5 (67.0)	254.5	5.4	0.4	17.3
15	Yes	30.1 (13.8)	45.0 (20.6)	143.3 (65.6)	218.4	33.6 (14.0)	42.3 (17.6)	164.3 (68.4)	240.2	3.5	−2.7	21.1
16	Yes	41.2 (17.4)	62.5 (26.4)	133.0 (56.2)	236.7	42.2 (16.2)	49.4 (19.0)	168.6 (64.8)	260.2	1.0	−13.0	35.7

*Note*: PCI = percutaneous coronary intervention.

**Table 4:** The electrophysiological changes at each location following PCI

	Difference (after – before PCI)													
	Collected point	P‐value	Dense Scar area, cm^2^	P‐value	Border Zone area, cm^2^	P‐value	Preserved Voltage area, cm^2^	P‐value	Low UNI area, cm^2^	P‐value	Normal UNI area, cm^2^	P‐value	LPs area, cm^2^	P‐value
All patients	−68 [−116 to 125]	0.75	1.0 [−1.6 to 3.7]	0.41	−6.6 [−13.6 to 0.4]	0.06	14.0 [2.7 to 25.2]	0.02	−6.9 [−15.2, 1.3]	0.14	15.3 [−3.4, 34.0]	0.13	−0.5 [−2.1, 1.2]	0.66
U‐group	130 [128 to 138]	0.0005	5.3 [−4.6 to 15.2]	0.15	9.9 [−17.3 to 37.1]	0.26	−8.7 [−47.8 to 30.4]	0.44	10.6 [−19.3, 40.5]	0.5	−18.6 [−37.0, 10.7]	0.5	−0.1 [−3.0, 2.7]	1
S‐group	−86 [−124 to −11]	0.09	0.1 [−2.8 to 2.9]	0.96	−10.5 [−16.8 to −4.1]	0.004	19.2 [7.7 to 30.6]	0.003	−11.0 [−19.0, −2.9]	0.02	23.1 [4.2, 42.0]	0.03	−0.5 [−2.6, 1.5]	0.62

*Note*: Numbers show the median difference [95% confidence interval]. LPs = late potentials; PCI = percutaneous coronary intervention; S‐group = patients with successful PCI and no restenosis; U‐group = patients with unsuccessful PCI or with reocclusion during the follow‐up period.

**Figure 3:**
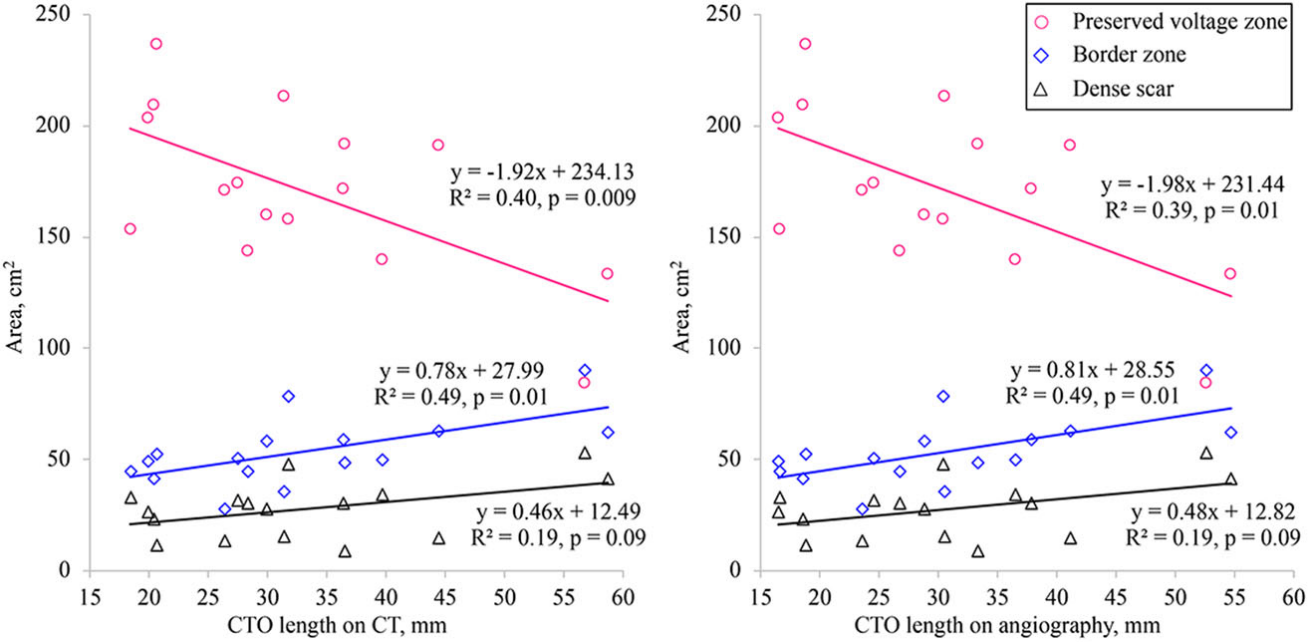
Correlation between the percent surface area of each voltage region and the CTO length measured by CT (left panel) or by coronary angiography (right panel). The percent area of the border zone showed a significant positive correlation with CTO length, while the percent area of the preserved voltage region had a significant negative correlation with CTO length. However, there was no significant correlation between the percent area of the dense scar region and CTO length. CT = computed tomography; CTO = chronic total occlusion [Color figure can be viewed at http://wileyonlinelibrary.com]

Figure 3 displays the correlations between area of each electrical region and the length of the CTO measured by CT or by coronary angiography. The area of the dense scar region did not show a significant correlation with CTO length. However, there was a significant positive correlation between the area of the border zone and CTO length, as well as a significant negative correlation between the area of the preserved voltage region and CTO length.

We also calculated the changes of unipolar and bipolar electrograms in the whole left ventricle (Table 5). Median unipolar and bipolar voltage amplitudes showed a slightly but significant increase in all subjects and the S‐group after PCI, while no significant voltage changes were observed in the U‐group.

**Table 5:** Changes of unipolar/bipolar voltage amplitude following coronary intervention

	Difference (after – before PCI)			
	Unipolar voltage amplitude, mV	P‐value	Bipolar voltage amplitude, mV	P‐value
All patients	1.29 [0.42, 2.99]	0.01	0.11 [0.03, 0.25]	0.01
U‐group	0.64 [−3.40, 1.56]	0.31	0.04 [−1.13, 1.13]	0.50
S‐group	1.55 [0.88, 3.33]	0.02	0.23 [0.08, 0.36]	0.004

*Note*: Numbers show the median difference (95% confidence interval). PCI = percutaneous coronary intervention; S‐group = patients with successful PCI and no restenosis; U‐group = patients with unsuccessful PCI or with reocclusion during the follow‐up period.

## 4 DISCUSSION

In this study, we evaluated the impact of successful recanalization of CTO on electrical remodeling of the left ventricle. To our knowledge, this is the first investigation of electrophysiological changes after successful PCI for CTO. Before PCI, the area of the dense scar region only showed a weak correlation with CTO length. However, there was moderate positive correlation between the area of the border zone and CTO length, as well as a moderate negative correlation between the area of the preserved voltage region and CTO length. Moreover, we found a significant decrease in the border zone area and the low UNI area after successful recanalization of CTO, while there was no change in the scar region. After successful PCI, the median unipolar and bipolar amplitudes of the whole left ventricle increased significantly, while no significant changes of unipolar and bipolar amplitudes were observed in patients with unsuccessful PCI.

There are differences among imaging modalities with respect to assessment and reporting of the extent and severity of myocardial ischemia and/or necrosis.^23^, ^24^ In patients with acute myocardial infarction, the extent of left ventricular remodeling is directly influenced by the area of myocardium impacted by coronary artery occlusion.^25^ In contrast, the low‐voltage zone may include a substantial amount of viable myocardium in patients with CTO and such myocardium may maintain electrophysiological viability. During a standard stress test with systemic infusion of adenosine, the coronary flow velocity and pressure changes distal to a site of occlusion are far below the cut‐off values for assessing the functional reserve in patients with nonocclusive lesions. Therefore, even well‐developed collaterals cannot prevent ischemia during exercise.^26^, ^27^, ^28^ Ladwiniec et al.^29^ reported that recanalization of a CTO leads to a moderate increase in the fractional flow reserve (FFR) of the predominant collateral donor vessel associated with reduction of coronary flow, and the border zone around the dense scar tissue may therefore be affected by changes in CTO length and collateral flow. Hence, the area of the border zone showed moderate correlation with CTO length while there was no significant correlation between the dense scar area and CTO length. It is possible that CTO could be associated with the extent of tissue necrosis. In addition, we investigated the correlationship between the percentage surface area of each voltage region and the CTO length and the each correlationship showed similar result (preserved voltage zone: y = –0.61x + 87.42, R^2^ = 0.43, P = 0.006; border zone: y = 0.38x + 8.74, R^2^ = 0.53, P = 0.01; dense scar zone: y = 0.22x + 3.85, R^2^ = 0.24, P = 0.053). The median value of the dense scar area did not change after intervention in the S‐group. On the other hand, the dense scar area trended to be larger in follow‐up, without reaching statistical significance. In addition, the cut‐off value (<0.5 mV) for dense scar might affect the result. Yoshida et al.^30^ reported the low voltage area (0.1–0.6 mV) could be useful for targeting catheter ablation. It means the dense scar less than 0.5 mV could include viable myocardium and successful PCI for CTO may affect the dense scar slightly as well as the border zone area. Moreover, the multipolar mapping catheter with 1.0‐mm electrodes was used for mapping in this study and the definitions of each region may be affected by the electrode size and inter‐electrode distance. Future validation study might be needed to identify the difference between EAM using 3.5‐mm or 4.0‐mm‐tip ablation catheter and EAM using 1.0‐mm electrodes. CTO of an infarct‐related artery may cause hypoperfusion around the necrotic zone that impacts the border zone and makes it more prone to ventricular arrhythmia.^5^ In patients with previous myocardial infarction, VT often occurs because of a scar‐related reentry circuit.^31^ In the border zone around the dense scar tissue, clumps of viable cardiomyocytes exist among fibrotic tissue, creating the slow conduction channels that are essential for reentry.^31^, ^32^ Further prospective studies will be required to determine whether recanalization of CTO can reduce the incidence of lethal ventricular arrhythmias.

The distribution of the substrate for VT depends on its etiology, with development of a VT substrate on the epicardial surface being common in nonischemic cardiomyopathy.^20^, ^33^, ^34^ On the other hand, ischemic cardiomyopathy is frequently associated with dysfunctional and heterogeneous endocardial conduction, while the need for epicardial access to treat VT is less frequent.^12^, ^13^ Several authors have reported reduction of the endocardial unipolar voltage in animal models of chronic infarction.^22^, ^35^ In our study, successful PCI for CTO had a modest but significant positive impact on not only bipolar voltage amplitude of the left endocardium but unipolar voltage amplitude. From previous research, while the bipolar signals are associated with local electrical activity, the unipolar electrograms are affected by distant activities like epicardial potentials.^36^ In addition, the endocardium unipolar electrogram could be more useful to distinguish transmural scar from noninfarct tissue and to detect the epicardium ventricular arrhythmia substrate.^22^, ^35^ Considering our results, recanalization of CTO may affect electrical remodeling on endocardium and epicardium. However, assessment of the epicardial bipolar voltage amplitude is a better method for determining electrophysiological changes accurately, but it is difficult to collect epicardial bipolar voltage data if there is no clinically necessary justification for accessing the epicardial space. Other noninvasive modalities like late gadolinium enhancement magnetic resonance imaging could possibly be useful to investigate changes in the epicardial substrate.

## 5 LIMITATIONS

Several limitations of this study should be taken into consideration. First, it was a single‐center study that enrolled a small number of patients. In addition, the U‐group included only three patients. However, these data compared the electrical remodeling before and after an intervention without any changes of medication except for clopidogrel. Second, electroanatomic mapping is operator‐dependent and may not be fully reproducible, even if performed by the same operator. If small differences are found in this study, it cannot be excluded that they are at least in part justified by mapping discrepancies. In addition, comparing each region at each ventricular segment might affect the result. However, dividing the segment according to electroanatomical map may be influenced by observer and introduce another bias. Moreover, as the voltage maps were obtained using two types of catheters with different electrode sizes and interelectrode spacing, errors may have been introduced in the detection of low‐voltage areas. However, the use of the 4‐mm tipped catheter was limited to situations where the multipronged catheter produced frequent ectopic activity. Third, we did not induce ventricular arrhythmia before or after PCI, and we have no data about the relationship between ventricular arrhythmia and reduction of the border zone area. Fourth, the follow‐up period was only about 8 months. While it is unclear whether this was long enough to evaluate the electrophysiological changes after PCI, Mohdnazri et al.^37^ reported that both FFR and the instantaneous wave‐free ratio were significantly increased in the territory of the predominant donor vessel after a follow‐up period of 4 months. Finally, the epicardial surface voltage was not measured directly, and we evaluated the unipolar signal amplitude as a surrogate for the epicardial voltage.

## 6 CONCLUSIONS

In this study population, the CTO length was not related to the area of the dense scar region, but had positive correlationship with the border zone area. Successful recanalization of a CTO may contribute to reverse electrical remodeling in the border zone of the left ventricular myocardium, but does not affect the dense scar region.
